# Prevalence and determinants of diabetes mellitus among Iranian patients with chronic liver disease

**DOI:** 10.1186/1472-6823-4-4

**Published:** 2004-11-19

**Authors:** Seyed M Alavian, Behzad Hajarizadeh, Fariborz Nematizadeh, Bagher Larijani

**Affiliations:** 1 Department of Internal Medicine, Baghiatollah University of Medical Sciences, Tehran, Iran; 2 Tehran Hepatitis Center, Tehran, Iran; 3 Endocrinology and Metabolism Research Center (EMRC), Tehran University of Medical Sciences, Tehran, Iran

## Abstract

**Background:**

Alterations in carbohydrate metabolism are frequently observed in cirrhosis. We conducted this study to define the prevalence of diabetes mellitus (DM) and impaired glucose tolerance (IGT) in Iranian patients with chronic liver disease (CLD), and explore the factors associated with DM in these patients.

**Methods:**

One hundred and eighty-five patients with CLD were enrolled into the study. Fasting plasma glucose and two-hour plasma glucose were measured in patients' sera. DM and IGT were diagnosed according to the latest American Diabetes Association criteria.

**Results:**

The subjects included 42 inactive HBV carriers with a mean age of 42.2 ± 12.0 years, 102 patients with HBV or HCV chronic hepatitis with a mean age of 41.2 ± 10.9 years, and 41 cirrhotic patients with a mean age of 52.1 ± 11.4 years. DM and IGT were diagnosed in 40 (21.6%) and 21 (11.4%) patients, respectively. Univariate analysis showed that age (P = 0.000), CLD status (P = 0.000), history of hypertension (P = 0.007), family history of DM (P = 0.000), and body mass index (BMI) (P = 0.009) were associated with DM. Using Multivariate analysis, age (OR = 4.7, 95%CI: 1.8–12.2), family history of DM (OR = 6.6, 95%CI: 2.6–17.6), chronic hepatitis (OR = 11.6, 95%CI: 2.9–45.4), and cirrhosis (OR = 6.5, 95%CI: 2.4–17.4) remained as the factors independently associated with DM. When patients with cirrhosis and chronic hepatitis were analyzed separately, higher Child-Pugh's score in cirrhotic patients (OR = 9.6, 95%CI: 1.0–88.4) and older age (OR = 7.2, 95%CI: 1.0–49.1), higher fibrosis score (OR = 59.5, 95%CI: 2.9–1211.3/ OR = 11.9, 95%CI: 1.0–132.2), and higher BMI (OR = 30.3, 95%CI: 3.0–306.7) in patients with chronic hepatitis were found to be associated with higher prevalence of DM.

**Conclusions:**

Our findings indicate that patients with cirrhosis and chronic hepatitis are at the increased risk of DM occurrence. Older age, severe liver disease, and obesity were associated with DM in these patients.

## Background

Alterations in carbohydrate metabolism are frequently observed in liver cirrhosis. Glucose metabolism impairment in cirrhotic patients, both in fasting state and in response to oral glucose or meals has been widely documented in the literature [1-5]. There is a wide variability in the prevalence of overt diabetes mellitus (DM) and impaired glucose tolerance (IGT) according to various reports. In fact, the laboratory methods and the criteria used to state glucose metabolism status are different among various studies, considering the fact that the criteria for diagnosing DM and IGT have been modified a couple of times during last decade. On the other hand, the majority of the papers focused on cirrhosis [2,3,6-10] although a number of studies evaluated patients with chronic hepatitis, as well [4,5,11,12]. Furthermore, in many studies investigating the correlation between liver disease and glucose tolerance, a number of potent factors for DM such as BMI have been overlooked.

In this study, for the first time in Iran, we reported the prevalence of DM and IGT in patients with chronic liver disease (CLD) using the latest generally accepted criteria, and investigated independent correlation of CLD with DM, considering other known possible DM risk factors. Moreover, we explored the factors that might be potentially associated with DM in patients with cirrhosis and chronic hepatitis.

## Methods

From October 2002 to March 2003, all consecutive patients with chronic liver disease referred to "Tehran Hepatitis Center" were enrolled into the study. Tehran Hepatitis Center is a referral specialized clinic for liver diseases where many patients suffering from liver diseases and hepatitis from around Iran are referred to in order to receive consultation and clinical medical care. Pregnant females, patients under 20 years old, those who were on regular corticosteroid or hydrochlorothiazide therapy, known or suspected cases of hemochromatosis, or autoimmune disease were excluded. Having a clinical, biochemical (serum amylase and/or lipase elevation) or ultrasonographic evidence for chronic pancreatitis was also considered an exclusion criterion. No patient had a history of habitual drinking. All of the patients were Moslem and belonged to the white race.

A total number of 193 patients who met the criteria were eligible to enter the study. First group included 41 cirrhotic patients. Mean age was 52.1 ± 11.4 years ranging between 26–74 years. Diagnosis of cirrhosis was confirmed by histology in 17 (41.5%) patients. The occurrence of signs or biochemical evidences of liver decompensation, ultrasound features of portal hypertension and/or esophageal varices in gastroscopy were used for the clinical diagnosis in the remaining cases. No cirrhotic patients had evidence of hepatocellular carcinoma, screened by serum alpha-fetoprotein level test and abdominal ultrasonography. Severity of liver cirrhosis was graded according to Child-Pugh's classification. Second group included 102 patients with chronic hepatitis B, or C. Mean age was 41.2 ± 10.9 years ranging between 22–67 years. All patients had adequate documentation of elevated serum aminotransferases for more than 6 months, and viral hepatitis molecular assays by qualitative RT-PCR. In 91 (89.2%) patients liver biopsy reports were accessible. The histological staging and grading in liver biopsy was scored according to Knodell scoring. Third group included 42 inactive hepatitis B virus (HBV) carriers who were hepatitis B s antigen (HBsAg) positive, quantitative HBV DNA level less than 10^5 ^copies/ml (ROCHE Cobas Amplicore) and normal liver enzymes. The mean age in this group was 42.2 ± 12.0 years ranging between 23–84 years. Eight patients with nonalcoholic steatohepatitis (NASH) were initially enrolled but were not entered in analysis because of the low case number. Therefore analysis was finally performed for 185 patients.

Informed consent in writing was obtained from each patient involved and the study protocol conformed to the ethical guidelines of the 1975 Declaration of Helsinki as approved by institutional review board. Initial data were collected by chart review and interview. Then, two venous blood samples were taken: First sample in the morning after a 12-hour overnight fast to measure fasting plasma glucose (FPG) level, triglycerides (TG) level, and cholesterol (Chol) level; and a second sample following 2 hours after eating 75 gr glucose to measure two-hour post-loaded glucose (2-hr PG). FPG and 2-hr PG measurement were repeated on two other samples in second session.

Patients receiving therapy with insulin or oral hypoglycemic medications were considered as diabetics. In other patients, FPG ≥ 126 mg/dl or 2-hr PG ≥ 200 mg/dl on more than one occasion was used as diagnostic for DM in agreement with the latest American Diabetes Association (ADA) criteria [13]. FPG between 110 mg/dl and 126 mg/dl was considered as impaired fasting glucose (IFG), and 2-hr PG between 140 mg/dl and 200 mg/dl was considered as IGT according to ADA definitions [13].

Body mass index (BMI) was calculated using the standard formula of weight in kilogram divided by square of height in meters (kg/m^2^).

Results were expressed as mean ± standard deviation (SD). Comparison between diabetic and non-diabetic groups was made using Students T test for continuous variables and the Chi-square or Fisher exact test for categorical variables. At univariate analysis the factors possibly associated with DM development were evaluated. A multivariate analysis based on a stepwise logistic regression model was used to assess the independent effect of all variables found significant at the univariate analysis. P-value less than 0.05 was considered significant. All statistical analyses were performed using SPSS for Windows software (version 10.0; SPSS Inc. Chicago, Illinois, USA).

## Results

The sample population contained 150 (81.1%) males and 35 (18.9%) females. The overall mean age was 43.8 ± 12.0 years ranging between 22 and 84 years. In the total of 185 patients, DM was found in 40 (21.6%) patients, and IFG and/or IGT was found in 21 (11.3%) patients. Thirty patients were already aware of their problem whereas the diagnosis of DM was new in 10 patients.

By univariate analysis a number of variables including the factors mentioned by ADA as risk factors for type II diabetes [13] in addition to status and etiology of liver disease, interferon therapy, and history of or ongoing habitual smoking were compared between diabetic and non-diabetic patients (Table 1). We found that the prevalence of DM was significantly associated with older age (P = 0.000), CLD status (P = 0.000), history of hypertension (P = 0.007), family history of DM (P = 0.000), and higher BMI (0.009). Excluding the inactive carriers, we compared DM rate in two other groups. The prevalence of DM among cirrhotic cases (53.7%) was significantly higher than that of chronic hepatitis patients (13.7%), (P = 0.000).

**Table 1:** Clinical and epidemiological characteristics of CLD patients with and without DM

	**Diabetic**	**Non-diabetic**	**P-value**
Number	40	148	
Sex			NS
Female	9 (22.5%)	26 (17.9%)	
Male	31 (77.5%)	119 (82.1%)	
Age	50.3 ± 11.4	40.2 ± 10.6	0.000
CLD status			0.000
Inactive carrier	4 (10.0%)	38 (26.2%)	
Chronic hepatitis	14 (35.0%)	88 (60.7%)	
Cirrhosis	22 (55.0%)	19 (13.1%)	
CLD etiology			NS
HBV	24 (60.0%)	85 (58.6%)	
HCV	13 (32.5%)	58 (40.0%)	
Cryptogenic	3 (7.5%)	2 (1.4%)	
Family history of DM*			0.000
Yes	21 (52.5%)	29 (20.0%)	
No	19 (47.5%)	116 (80%)	
History of hypertension^†^			0.007
Yes	10 (25.0%)	13 (9.0%)	
No	30 (75.0%)	132 (91.0%)	
BMI	27.5 ± 4.6	24.7 ± 4.0	0.009
Interferon therapy			NS
Yes	6 (15.0%)	49 (33.8%)	
No	34 (85.0%)	96 (66.2%)	
Smoking			NS
Yes	13 (32.5%)	37 (25.5%)	
No	27 (67.5%)	108 (74.5%)	
TG	179.2 ± 74.1	142.8 ± 71.4	NS
Chol	192.4 ± 39.8	174.7 ± 44.4	NS

*Indicates presence of at least one first-degree relative affected by DM.

^†^Includes patients who require diet or antihypertensive agents.

At multivariate analysis age (P = 0.008, OR = 4.7, 95%CI: 1.8–12.2), family history of DM (P = 0.000, OR = 6.6, 95%CI: 2.6–17.6), chronic hepatitis (0.000, OR = 11.6, 95%CI: 2.9–45.4), and cirrhosis (P = 0.000, OR = 6.5, 95%CI: 2.4–17.4) showed up as the only factors independently associated with DM prevalence rate (table 2).

**Table 2:** Logistic regression analysis of factors associated with DM among patients with CLD

	**OR**	**95% CI**	**P-value**
Age			
< 45 years	1.0		
≥ 45 years	4.7	1.8 – 12.2	0.001
CLD status			
Inactive carrier	1.0		
Chronic hepatitis	11.6	2.9 – 45.4	0.000
Cirrhosis	6.5	2.4 – 17.4	0.000
Family history of diabetes			
No	1.0		
Yes	6.6	2.6 – 16.7	0.000
History of hypertension			
No	1.0		
Yes	2.3	0.7 – 8.1	NS
BMI			
> 25	1.0		
≥ 25	1.4	0.5 – 3.4	NS

Since both cirrhosis and chronic hepatitis were found as independent factors associated with DM occurrence, we analyzed these two groups separately. The results were summarized in table 3.

**Table 3:** Clinical and epidemiological characteristics of patients with and without DM separated to cirrhosis and chronic hepatitis groups

	**Patients with cirrhosis**			**Patients with chronic hepatitis**		
	
	**Diabetic**	**Non-diabetic**	**P-value**	**Diabetic**	**Non-diabetic**	**P-value**
Number	22	19		14	88	
Sex			NS			NS
Female	4 (18.2%)	3 (15.8%)		2 (14.3%)	12 (13.6%)	
Male	18 (81.8%)	16 (84.2%)		12 (85.7%)	76 (86.4%)	
Age	56.9 ± 10.6	46.6 ± 10.0	0.003	49.7 ± 7.8	39.9 ± 10.7	0.001
CLD etiology			NS			NS
HBV	11 (50.0%)	13 (68.4%)		10 (71.4%)	37 (42.0%)	
HCV	8 (36.4%)	4 (21.1%)		4 (28.6%)	51 (58.0%)	
Criptogenic	3 (13.6%)	2 (10.5%)		-	-	
Family history of DM			NS			0.01
Yes	9 (40.9%)	3 (15.8%)		8 (57.1%)	21 (23.9%)	
No	13 (59.1%)	16 (84.2%)		6 (42.9%)	67 (76.1%)	
History of hypertension			NS			NS
Yes	6 (27.3%)	1 (5.3%)		3 (21.4%)	8 (9.1%)	
No	16 (72.7%)	18 (94.7%)		11 (78.6%)	80 (90.9%)	
BMI	26.4 ± 3.4	26.4 ± 4.0	NS	27.7 ± 4.8	24.5 ± 3.1	0.02
Interferon therapy			NS			NS
Yes	2 (9.1%)	3 (15.8%)		4 (28.6%)	46 (52.3%)	
No	20 (90.9%)	16 (84.2%)		10 (71.4%)	42 (47.7%)	
Smoking			NS			NS
Yes	5 (22.7%)	5 (26.3%)		7 (50.0%)	25 (28.4%)	
No	17 (77.3%)	14 (73.7%)		7 (50.0%)	63 (71.6%)	
TG	145.5 ± 58.6	109.3 ± 75.2	NS	174.9 ± 82.5	140.6 ± 53.8	NS
Chol	141.6 ± 50.5	151.8± 32.5	NS	199.8 ± 42.8	163.9 ± 36.1	NS
Child-Pugh's score			0.04			
Score A	13 (65.0%)	18 (94.7%)		-	-	-
Score B	7 (35.0%)	1 (5.3%)				
Histological staging*						0.003
0–1	-	-	-	1 (8.3%)	33 (41.8%)	
2–3				3 (25.0%)	30 (38.0%)	
4–6				8 (66.7%)	16 (20.3%)	
Histological grading*						NS
0–4	-	-	-	2 (16.7%)	36 (45.6%)	
5–8				8 (66.7%)	34 (43.0%)	
≥ 9				2 (16.7%)	9 (11.4%)	

* According to Knodell score

Out of 41 cirrhotic patients, 22 (53.7%) patients were diabetic and 7 (17.1%) patients had IFG and/or IGT. Univariate analysis showed that only two factors were associated with DM rate. Mean of age in diabetic cases was significantly higher than that of non-diabetic ones (P = 0.003). Moreover, DM was significantly more prevalent in patients with Child-Pugh's score B than score A (P = 0.04). Since there was no score C in study sample, we could not evaluate this class of cirrhosis. After applying logistic model, only Child-Pugh's score kept its significance (P = 0.04, OR = 9.6, 95%CI: 1.0–88.4) as an independent predictive factor for DM in cirrhotic patients (table 4).

**Table 4:** Logistic regression analysis of factors associated with DM among cirrhotic patients and patients with chronic hepatitis.

	**OR**	**95% CI**	**P-value**
**Cirrhosis**			
Age			
< 45 years	1.0		
≥ 45 years	2.1	0.4 – 10.5	NS
Child Pugh's Score			
Score A	1.0		
Score B	9.6	1.0 – 88.4	0.04
**Chronic hepatitis**			
Age			
> 45 years	1.0		
≥ 45 years	7.2	1.0 – 49.1	0.04
Family history of diabetes			
No	1.0		
Yes	2.0	0.3 – 13.9	NS
BMI			
> 25	1.0		
≥ 25	30.3	3.0 – 306.7	0.004
Histological staging			
0–1	1.0		
2–3	59.5	2.9 – 1211.3	0.008
4–6	11.9	1.0 – 132.2	0.04

In chronic hepatitis group including 102 patients, DM was found in 14 (13.7%) patients and IFG and/or IGT was found in 11 (10.8%) patients. There was no significant difference between diabetic and non-diabetic cases in regard with sex, etiology of chronic hepatitis, hypertension, interferon therapy, smoking, and serum TG and Chol levels. On the other hand, diabetic patients had higher mean age compared with non-diabetic cases (P = 0.001). More cases of diabetic patients compared with non-diabetic ones had a family history of DM (P = 0.01). The mean BMI of patients with DM was higher than that of patients without DM (P = 0.02). Furthermore, liver biopsy showed significantly more fibrosis activity in diabetic patients compared with non-diabetic cases (P = 0.003), whereas no difference in diabetic vs. non-diabetic patients was seen with respect to histological grading. Multivariate analysis revealed that older age (P = 0.04, OR = 7.2, 95%CI: 1.0–49.1), higher BMI (P = 0.004, OR = 30.3, 95%CI: 3.0–306.7), and more severe fibrosis activity (stage 2–3: P = 008, OR = 59.5, 95%CI: 2.9–1211.3; stage 4–6: P = 0.04, OR = 11.9, 95%CI: 1.0–132.2) were the predictive variables for DM in patients with chronic hepatitis (table 4).

## Discussion

There is a wide range in the prevalence of glucose metabolism alterations in cirrhotic patients in various studies. The frequency for overt DM has been reported from 10% to 50% and for IGT up to more than 70% [1-5,7,8,10,11]. Such variations may be mainly due to the criteria employed in the diagnosis of DM. In our study, we employed latest ADA criteria and DM was presented in 21.6% of patients with CLD (53.7% in cirrhosis, 13.7% in chronic hepatitis, and 9.5% in HBV inactive carrier). In Iran the estimated prevalence of overt DM in general population is in the range of about 7.5–10 percent [14-16], which is about 2 to 3 times less than what we found in this study, indicating that patients with CLD are a high-risk population for DM. In spite of this fact, we showed that 10 out of 40 (25%) diabetic cases found in this study were unaware of their endocrinal problem before being screened as part of this study. This finding highlights the importance of periodical screening of the patients with CLD especially in advanced stages.

Literature frequently demonstrated the higher prevalence of DM and IGT in cirrhosis than in chronic hepatitis [4,5,11]. However, about the higher DM rate in patients with chronic hepatitis compared with people without liver disease, there is not any widely general agreement. Some studies claimed that DM rate is not appreciably different when compared with general population or individuals without liver disease [11,17]. These studies believed that only cirrhosis but not chronic hepatitis were associated with DM [17]. In our study, multivariate analysis indicates independent association between chronic hepatitis and DM rate, despite the fact that we compared DM occurrence among three groups who all suffered from liver disease. Moreover, our data indicate that the frequency of DM increases significantly with the severity of the liver disease both in cirrhotic cases and in patients with chronic hepatitis. These findings suggest that liver fibrosis but not cirrhosis itself, is the event associated with glucose intolerance. A weak association between glucose intolerance and severity of liver disease in cirrhosis has already been reported by Muller et al [7], even though they used different criteria to evaluate liver damage severity. Another study applying Child-Pugh's classification showed similar results [11]. In this context it is worthy to note that although the underlying mechanism of glucose intolerance in cirrhosis has not been fully elucidated, it can be mainly explained by the insulin resistance [9], in addition to reduced glucose sensitivity in liver cirrhosis [18,19]. More interestingly, a recent study suggests that insulin resistance occurs already in the early stages of the chronic hepatitis course [12], and another study investigating chronic hepatitis patients with normal glucose tolerance revealed a strong relationship between insulin sensitivity and fibrosis score [20]. In contrast, there is another hypothesis that indicates CLD as a consequence of DM. This theory states that occurrence of insulin resistance initially facilitates lipolysis, and increases free fatty acid deposition in liver, which increases products of lipid peroxidation inducing oxidative stress. This results in cytokine-mediated hepatic inflammatory damage that induces collagen deposition and eventually fibrosis [21,22]. Although in this study we could not determine if the onset of DM was before or after of liver disease, our findings show that chronic hepatitis per se has an independent association with DM, and development of DM in chronic hepatitis patients was correlated to the severity of liver fibrosis.

No differences in DM rate were found when we studied patients according to CLD etiological categories both in cirrhosis and in chronic hepatitis group. A positive link between hepatitis C infection and development of DM has been suggested in some studies but not completely characterized. Mason et al surveying a large cohort of patients with viral chronic hepatitis showed a relatively strong association between DM and HCV infection [23]. They suggested that HCV infection may serve as an additional risk factor for the development of DM beyond that attributable to CLD alone [23]. In contrast, some studies provided evidence against a potential association between these two disorders [17,24]. A more recent study showed that the prevalence of DM among cirrhotic patients with hepatitis C was significantly higher than among those with cirrhosis due to cholestatic liver disease, but this rate difference was not significant when compared with cases with alcohol-induced cirrhosis [10]. In spite of the fact that they had not enrolled HBV infected cases in their sample population, it suggested that the mechanism of the DM in cirrhotic patients was related more closely to the underlying cause of liver cirrhosis [10]. Although no single etiological factor was found linked to DM occurrence in our study, we believe that our study was not designed to address this issue considering the fact that we had to exclude patients with NASH as another etiology of CLD because of small number of patients in this group. However, further prospective studies may shed more light on the relationship between DM and the underlying liver disease.

BMI did not differ between diabetic and non-diabetic cases among cirrhotic patients. This finding was expectable as reduction in the muscle mass is well described in liver cirrhosis, which can bias the comparison. On the other hand, among patients with chronic hepatitis BMI did remain as an independent predictive factor of DM. Some studies have indicated that obesity could be a potential risk factor for fibrosis in chronic hepatitis [25]. Our results suggest that increased BMI also has a role in the pathogenesis of DM in chronic hepatitis independent of liver fibrosis. Furthermore, Muller et al reported that basal free fatty acids and basal free glycerol plasma concentrations were increased in diabetic patients with liver cirrhosis when compared with those without diabetes [7]. In present study, TG and Chol levels in patients with chronic hepatitis and TG level in cirrhotic patients were higher in diabetic cases compared with non-diabetic ones although not statistically significant (table 3). These findings may have therapeutic implications in the management of patients with chronic hepatitis. It appears necessary to greatly encourage overweight patients to make a concerted effort to lose weight in order to both decrease the risk of DM development and to prevent the liver damage.

Although family history of DM is a well-known risk factor for DM, the correlation between DM and both cirrhosis and chronic hepatitis remained significant even when family history of DM was entered through logistic model. This finding, in concordance with similar studies [7,11], indicates that liver injury per se is associated with DM and a family history of DM is only an adjunctive factor. However, the possible reporting bias of family history of DM should be carefully considered as it is not unreasonable to think that patients with known DM may be more likely to know or think that they have a family history of DM. When cirrhosis and chronic hepatitis groups were analyzed separately, we observed that patients with a positive family history of DM did not show an increased frequency of DM, particularly in cirrhotic patients. Slightly less than 45% of cirrhotic cases with a negative family history of DM were diabetic. This finding does not support the speculation that cirrhotic patients only with a genetic predisposition for DM are prone to glucose intolerance as the manifestation of their liver disease.

Age is another definite risk factor for type II DM in the normal population [13], and it was expected also to be associated with DM in CLD. However, the odds ratio for both cirrhosis and chronic hepatitis was higher than that for age, implying a stronger association of the former factors. Although in the part of our study investigating cirrhotic patients, age lost its significance when it was entered in multivariate analysis, the majority of studies demonstrated that IGT and also DM were more frequently seen at advanced age in cirrhotic patients [2,7,11].

While DM was associated with hypertension in univariate analysis (table 1), in multivariate analysis it lost its significance as a predictor of DM (table 2). These two variables may both be related to the metabolic syndrome and adjustment for other related variables removes this association. On the other hand, it is currently believed that cardiovascular disease is rare in cirrhosis and studies demonstrate that cirrhotic patients, even in the presence of overt DM, have a low prevalence of vascular disease including hypertension [26]. Our findings support this hypothesis showing no difference in the rate of hypertension between diabetic and non-diabetic patients.

A few patients have so far been reported to develop DM during interferon therapy [27], but the evidences are insufficient in this regard as yet. In our study, interferon therapy had no impact on the prevalence of DM, since 15% of subjects with DM and about 39% without had a recorded use of interferon, and the difference was not significant. This is in line with some previous studies clarifying the impact of long-term administration of interferon on glucose metabolism [28].

## Conclusions

In summery, the present study indicates a high prevalence of DM in patients with CLD in Iran although the relatively small number of patients in each of the three subgroups particularly cirrhosis and inactive carriers was a major shortcoming of our study and may have potentially underestimated or overestimated the prevalence of DM in these subgroups of patients. Since a considerable number of diabetic patients were unaware of their problem, it is imperative that the patients be screened for glucose intolerance periodically. For more severe stages of liver disease the screening interval appears to be shorter because of higher probability for DM occurrence. Furthermore, our findings show that weight reduction for overweight patients with chronic hepatitis is of benefit in order to prevent the occurrence of DM.

## List of abbreviations

DM: Diabetes Mellitus; IGT: Impaired Glucose Tolerance; CLD: Chronic Liver Disease; BMI: Body Mass Index; HBV: Hepatitis B virus; HBsAg: Hepatitis B s Antigen; NASH: Nonalcoholic Steatohepatitis; FPG: Fasting Plasma Glucose; TG: Triglycerides; Chol: Cholesterol; 2-hr PG: Two-hour Post-loaded Glucose; ADA: American Diabetes Association; IFG: Impaired Fasting Glucose.

## Competing interests

The author(s) declare that they have no competing interests.

## Authors' contributions

Dr. Alavian participated in the design of the study and coordination and drafted the manuscript. Dr. Hajarizadeh conceived of the study, participated in the design of the study, performed the statistical analysis and drafted the manuscript. Dr. Nematizadeh designed the questionnaire and helped in data collection. Dr. Larijani facilitated the study progress and participated in coordination. All authors read and approved the final manuscript.

## Pre-publication history

The pre-publication history for this paper can be accessed here:


